# 7-T MRI-based surrogate for histopathology examination of liver fibrosis

**DOI:** 10.1186/s41747-025-00589-8

**Published:** 2025-05-23

**Authors:** Jérémy Dana, Antonin Fattori, Chrystelle Po, Aurélie Beaufrère, Valérie Vilgrain, Valérie Paradis, Patrick Pessaux, Thomas F. Baumert, Benoît Gallix, Aïna Venkatasamy

**Affiliations:** 1 Université de Strasbourg, Inserm, UMRS 1110, Institute for Translational Medicine and Liver Disease, Strasbourg, France; 2 Institute of Image-Guided Surgery - IHU Strasbourg, Strasbourg, France; 3 Department of Diagnostic Radiology, McGill University, Montreal, Canada; 4 Augmented Intelligence & Precision Health Laboratory (AIPHL), Research Institute of the McGill University Health Centre, Montreal, Canada; 5 Département de Pathologie, Hôpitaux Universitaires de Strasbourg, Strasbourg, France; 6 ICube UMR 7357, Université de Strasbourg/CNRS, Fédération de Médecine Translationnelle de Strasbourg, Strasbourg, France; 7 Département de Pathologie, Hôpital Beaujon, FHU MOSAIC, Université Paris Cité, Paris, France; 8 Département de Radiologie, Hôpital Beaujon, Université Paris Cité, Paris, France; 9 Service de Chirurgie Viscérale et Digestive, Nouvel Hôpital Civil, Hôpitaux Universitaires de Strasbourg, Strasbourg, France; 10 Pôle Hépato-digestif, Service d’Hépatogastroenterologie, Hôpitaux Universitaires de Strasbourg, Strasbourg, France; 11 Département de Radiologie, Hôpital Américain de Paris, Paris, France; 12 Inria, Institut national de recherche en sciences et technologies du numérique, Paris, France; 13 Plateforme Imageries du Vivant, Université de Paris, PARCC, INSERM, Paris, France; 14 Department of Radiology–Medical Physics, University Hospital Freiburg, Freiburg, Germany

**Keywords:** Hepatitis, Histopathology, Liver diseases, Liver fibrosis, Magnetic resonance imaging

## Abstract

**Background:**

To demonstrate that 7-T magnetic resonance imaging (MRI) provides a surrogate for histopathology of fresh *ex vivo* liver tissue, using the case study of liver fibrosis.

**Methods:**

We prospectively enrolled 20 patients undergoing surgical liver resection between November 2021 and April 2023. Each *ex vivo* fresh liver tissue specimen (~ 1 cm^3^) was sectioned in half. The first half, stained using Masson’s Trichrome and Perls, was assessed by three pathologists using the METAVIR score (reference standard). The second half was imaged with 7-T MRI using a cryoprobe (fat-suppressed T2-weighted turbo/fast spin-echo sequence, spatial resolution 75 × 75 × 200 µm^3^) and assessed by three radiologists and the same three pathologists, using a newly developed MRI-METAVIR score.

**Results:**

Five patients were excluded from the final analysis (one patient due to poor specimen quality, two due to surgery cancellation, and two previously published used for reader training). Of the remaining 15 patients, 10 (67%) presented with chronic liver diseases and 8/15 (53%) with advanced (F3 or F4) fibrosis. Radiologists achieved 88% sensitivity, 100% specificity, 93% accuracy (95% confidence interval 68–100%) and 0.94 Harrell’s c-index (0.86–1.00). Pathologists achieved 88% sensitivity, 86% specificity, 87% accuracy (60–98%) and 0.87 Harrell’s c-index (0.74–0.99). There were no statistically significant differences between MRI-based and pathologic reference standard stage (*p ≥* 0.655).

**Conclusion:**

With an in-plane spatial resolution of ~ 75 × 75 µm^2^, MRI paralleled low-magnification histology, enabling the assessment of micro-architectural liver changes, and provided a surrogate for histopathology examination of fresh *ex vivo* liver tissue samples at a microscopic level.

**Relevance statement:**

7-T MRI provides a surrogate for histopathology visualisation of fresh *ex vivo* liver tissue, opening new research perspectives for clinical high-field MRI of the liver.

**Key Points:**

- Using the newly developed MRI-METAVIR score, 7-T MRI data strongly correlated with histopathology, achieving excellent agreement and accuracy.
- 7-T MRI accurately differentiated advanced from minimal liver fibrosis.
- 7-T MRI visualises liver micro-architecture, enabling pathology-like, noninvasive three-dimensional imaging.

**Graphical Abstract:**

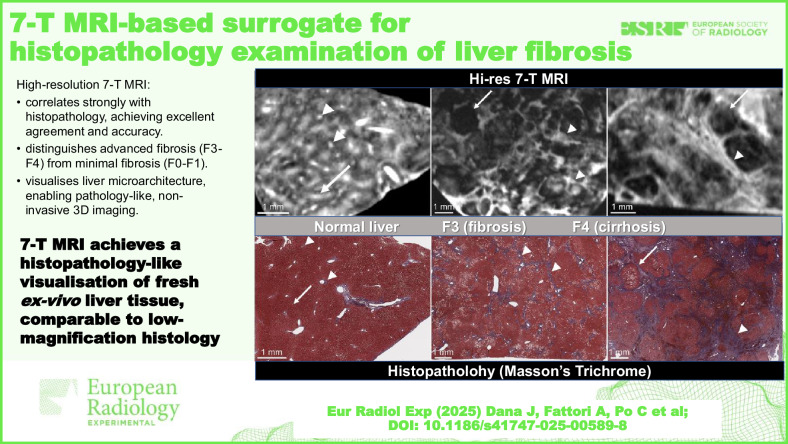

## Background

Histopathological examination, the standard of care for tissue analysis, requires intricate technical processes for tissue preparation, which inevitably delay the availability of results by at least 12 h following tissue sampling, rendering it unsuitable for intraoperative decision-making [1]. Extemporaneous histopathological examination, which employs an alternative technical procedure, enables a reduction in turnaround time, yielding preliminary results in less than 30 min [2]. However, these alternative procedures involve a freezing step and are responsible for tissue alterations such as morphological artefacts and sample exhaustion. Additionally, histopathological examination of liver biopsies is also prone to sampling errors [3] and/or inter-reader variability [4]. Although rare, liver biopsies are invasive with risks of bleeding complications [5].

High-field magnetic resonance imaging (MRI), with recent improvements such as radiofrequency coils, sequence optimisation, and imaging postprocessing [6], is now able to reach a very high spatial resolution, approximating that of histology (*i.e*., < 100 µm, compared to ~ 1 mm for clinical MRI) and appears as a promising tool to image fresh *ex vivo* tissues [7, 8]. Several studies reported the potential of high-field MRI for a pathology-like analysis of resected breast and lymphatic tissue on fresh *ex vivo* tissue samples [7, 9]. Similarly, the potential of this tool to provide pathology-like images was previously confirmed on two *ex vivo* liver samples [8]. Additionally, MRI is a noninvasive imaging modality, without the incompressible tissue processing time required for histology, with limited tissue preparation. High-field MRI appears as a promising “missing link” between conventional imaging and histology.

Liver fibrosis is characterised by the accumulation of extracellular matrix proteins caused by sustained liver insults. It is the inevitable consequence of all progressive chronic liver disease, ultimately leading to cirrhosis with complications such as hepatocellular carcinoma and portal hypertension. Liver fibrosis appears as an appropriate model for exploring high-resolution MRI because of the existence of widely validated histological classifications of liver fibrosis, with low subjectivity, such as the METAVIR system score used to classify the liver tissue on Masson’s trichrome-stained sections into five levels, from F0 to F4 [10–15].

This study aims to demonstrate that high-field MRI provides an accurate surrogate for histopathology examination of fresh *ex vivo* tissue using the case study of liver fibrosis on surgical liver samples.

## Methods

This prospective project was approved by the Research Ethics Board (Protocol RIPH2, LivMod N°IDRCB 2019-A00738-49 ClinicalTrial NCT04690972) and followed ethical principles of the Declaration of Helsinki. All patients provided written informed consent.

### Study design

**Fig. 1:**
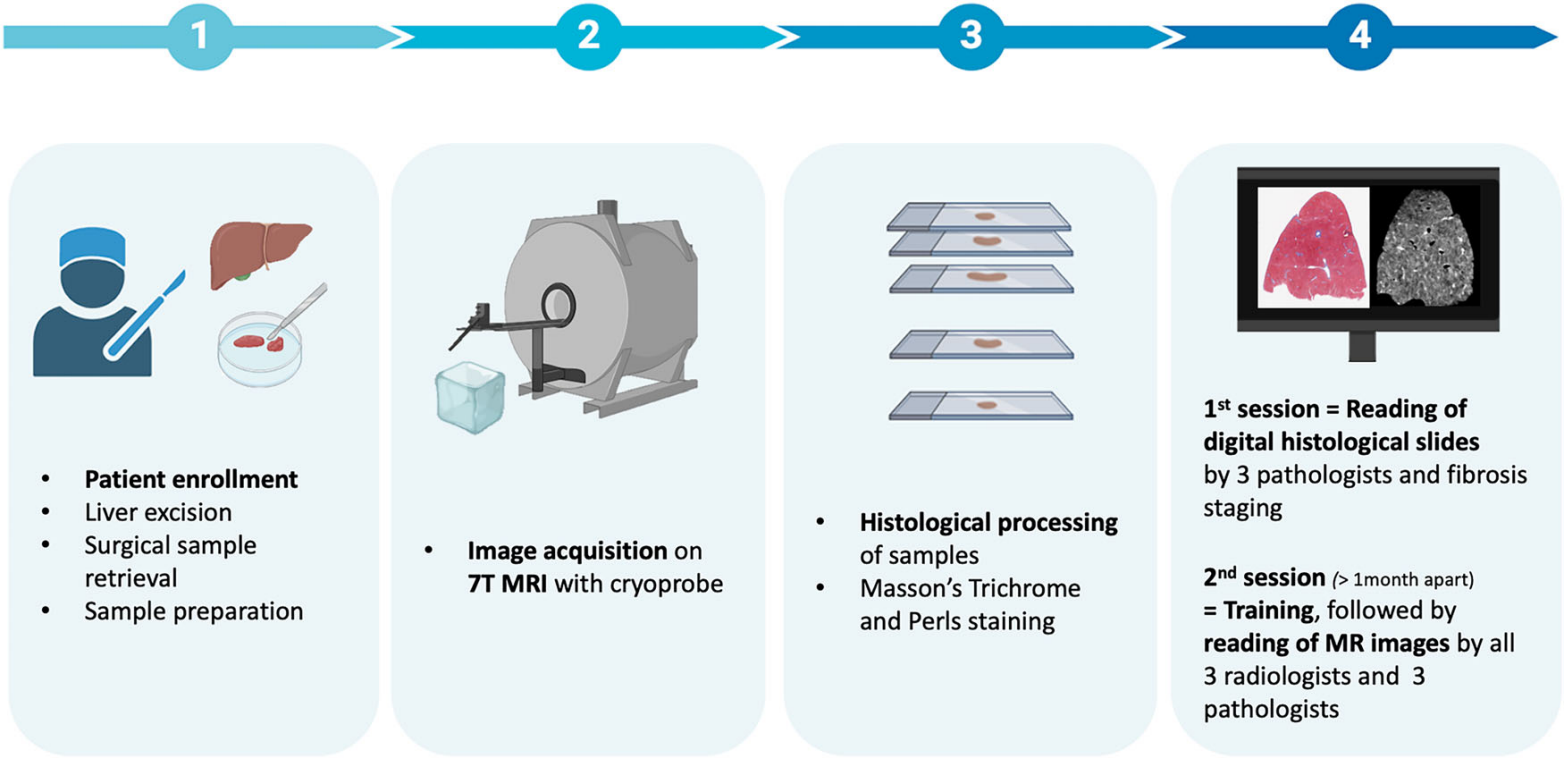
Schematic diagram of the overall workflow of the study

This prospective study enrolled twenty patients aged > 18 years, who underwent surgical liver resection between November 2021 and April 2023. The overall study workflow is presented in Fig. 1. We used *ex vivo* fresh liver tissue (~ 1 cm^3^) from surgically resected livers. Each fragment was sectioned in half, and the sections were identified, so that the MRI acquisition plane was as close as possible to the histological one. We imaged the first half, placed in Fluorinert™ Electronic Liquid FC40 (Sigma-Aldrich, Overijse, Belgium), a nonconductive, thermally and chemically stable fluid without ^1^H signal, using a 7-T MRI system with a cryoprobe (Bruker BioSpin, Rheinstetten, Germany). T2-weighted fat-suppressed turbo/fast spin-echo images were acquired with a rapid imaging with refocused echoes (RARE) sequence, whose parameters are summarised in Table 1. The cryoprobe technology employs cryogenically cooled radiofrequency coils and amplifiers cooled by a closed-cycle refrigeration system, allowing the reduction of electronic noise. The second half was fixed in formalin, embedded in paraffin, cut at a 4-µm thickness in the same plane as the MRI acquisition, and then stained using Masson’s Trichrome and Perls. The minimum processing time for pathology samples was around 48 h. All histology slides were digitally scanned using the Ventana DP 200 slide scanner (Roche, Basel, Switzerland). The low magnification used ranged between 1× and 2×, depending on the sample, and the scale bar was indicated for each microphotograph.

**Table 1:** Sequence parameters for the rapid imaging with refocused echoes (RARE) T2-weighted sequence

Repetition time (ms)	Echo time (ms)	Number of excitations	RARE factor	Matrix	Slice thickness (µm)	Field-of-view (mm)	Spatial resolution (µm)	Acquisition time (min)
3,000	30	25	2	266 × 200 × 42	200	20 × 15 × 6	75 × 75 × 200	240

### Assessment of MRI and pathology images

Three subspecialty-trained abdominal radiologists (B.G., V.V., and A.V., with 27, 35, and 7 years of experience, respectively) and three subspecialty-trained abdominal pathologists (A.F., V.P., and A.B. with 5, 30, and 10 years of experience) assessed both MRI and pathology images, independently and blinded to medical records. The readings of MRI and pathology images were conducted in two separate sessions, over a month apart.

#### Pathology images

In the first session, the three pathologists independently staged fibrosis on digital histological slides according to the METAVIR score using the software QuPath® [16]. The consensus fibrosis stage for pathology was based on the agreement of at least two of the three pathologists.

#### MRI

**Fig. 2:**
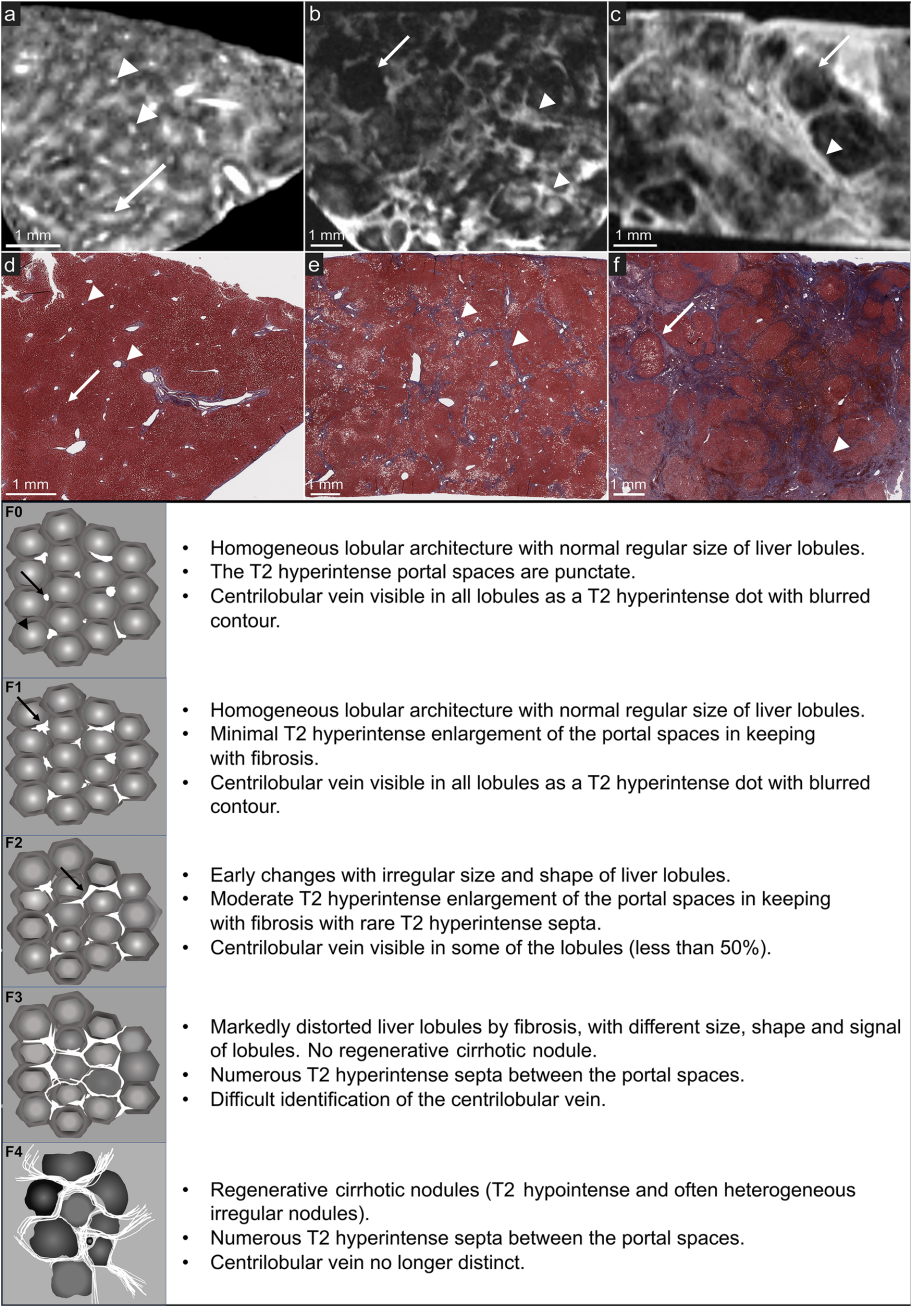
Schematic illustrations of the MRI-METAVIR score with high-resolution liver magnetic resonance imaging and histopathology correlation (Masson’s Trichrome) in a normal liver (**a**, **d**), F3 liver T2 (**b**, **e**), and F4 (cirrhosis) liver (**c**, **f**). On **a** and **d**, the liver architecture is homogeneous with hyperintense punctate portal spaces (arrow) and centrilobular veins (arrowhead). On **b** and **e**, the liver architecture is distorted by numerous T2 hyperintense fibrotic septa (arrowhead) with lobules of different size, shape, and signal (arrow). On **c** and **f**, there are regenerative cirrhotic nodules (arrow) separated by fibrotic septa (arrowhead)

The second session consisted of the reading of MRI images using 3D Slicer [17], the cases being presented in a different random order, by all three radiologists and three pathologists, given that this new field of application is halfway between the two specialities. First, all readers (radiologists and pathologists) received training on the METAVIR classification of fibrosis and the semiology of the fibrosis on high-field MRI (MRI-METAVIR score as described below), using two previously published cases [8] and schematic illustrations of high-field MRI for each stage (F0 to F4, Fig. 2). Then, all reviewers were asked to review the MRI images (6 images per patient) and stage fibrosis, according to the MRI-METAVIR score. The consensus fibrosis stage for MRI was based distinctively on the agreement of at least 2 two of the three radiologists and on the agreement of at least two of the three radiologists. To create the MRI-METAVIR score, one subspecialty-trained abdominal radiologist who did not participate in the reading (J.D., with 3 years of experience) reviewed all MRI images and analysed the MRI features of fibrosis in correlation with their corresponding histological section. The MRI-METAVIR score has been developed in analogy with the histological staging criteria, based on the presence and distribution of fibrosis [8].

### Reference standard

The reference standard was the consensus fibrosis METAVIR stage based on the agreement of at least two of the three pathologists using the pathology images only.

### Endpoint

The endpoint of the study was the performance of 7-T MRI to diagnose advanced fibrosis (stage F3 or F4) in fresh *ex vivo* liver tissue using the newly developed MRI-METAVIR score.

### Statistical analysis

Performance metrics of MRI were computed for sensitivity, specificity, accuracy, positive and negative predictive values, area under the receiver operating characteristic curve, and c-index. Paired ordinal non-normally distributed data were compared with the Wilcoxon signed-rank test. The concordance between the pathologic gold standard and the MRI consensus was calculated using weighted κ (linear weights). The inter-reader agreement was calculated using Fleiss κ [18]. The agreement was interpreted according to κ values as follows: < 0.00 (poor); 0.00–0.20 (slight); 0.21–0.40 (fair); 0.41–0.60 (moderate); 0.61–0.80 (substantial); 0.81–1.00 (almost perfect) [19]. All statistical analysis was performed using MedCalc Statistical Software version 23.0.5 (MedCalc Software bv, Ostend, Belgium; https://www.medcalc.org) and SPSS Statistics version 29 (IBM, IBM Corporation, New York, USA).

## Results

### Population

A total of 20 consecutive patients scheduled for liver surgery were included in the study. There was no adverse event or complication during the specimen collection or MRI procedure. Three patients were excluded due to poor specimen quality, not allowing MRI or pathology examination (*n* = 1) and cancellation of the surgery (*n* = 2). In addition, two of the 20 included patients, previously published [2], were used for reader training and not included in the final dataset. The final dataset consisted of 15 patients, 12 men and 3 women, with a median age of 68 years, interquartile range 60–73, who underwent liver surgery for primary liver lesions (*n* = 10) or colorectal cancer liver metastases (*n* = 5). Chronic liver disease of several aetiologies was present in 10 of 15 patients (67%) (Table 2).

**Table 2:** Demographics, clinical characteristics, surgical indications, and results of consensus review of pathology slides and 7-T magnetic resonance imaging fibrosis staging

Age (years)	Sex	Chronic liver disease	Surgical indication	Pathological fibrosis stage	MRI fibrosis stage
59	M	Hepatitis B and C viruses	Hepatocellular carcinoma	F2	Training
49	F	None	Metastases	F0	Training
73	M	MASLD	Hepatocellular carcinoma	F1	F0
57	M	Indeterminate aetiology	Metastases	F3	F2
73	M	None	Metastases	F0	F0
64	M	MetALD (MASLD and increased alcohol intake)	Hepatocellular carcinoma	F3	F3
58	M	None	Biliary cystadenoma	F1	F1
65	M	Alcohol associated liver disease	Hepatocellular carcinoma	F4	F4
73	M	MetALD (MASLD and increased alcohol intake)	Hepatocellular carcinoma	F1	F1
72	F	Alcohol associated liver disease	Hepatocellular carcinoma	F3	F4
71	M	None	Metastases	F1	F0
62	M	Alcohol associated liver disease	Hepatocellular carcinoma	F4	F4
56	F	None	Metastases	F0	F0
68	M	Indeterminate aetiology	Hepatocellular carcinoma	F3	F3
60	F	None	Metastases	F1	F1
68	M	Alcohol associated liver disease	Hepatocellular carcinoma	F3	F4
80	M	MASLD	Hepatocellular carcinoma	F4	F4

*F* Female, *M* Male, *MASLD* Metabolic dysfunction-associated steatotic liver disease

The pathologists’ consensus results for fibrosis staging on Masson’s Trichrome-stained slides, which served as a reference standard, were as follows: 2 of 15 patients (13%) had a METAVIR stage of F0, 5 (33%) of F1, 5/15 (33%) of F3, and 3/15 (20%) of F4 (Table 1). In all cases, no consensus review was necessary, and the agreement between the pathologists regarding the review of histopathology images was very good (κ = 0.77, 95% confidence interval (CI) 0.61–0.93).

### Accuracy of MRI

**Fig. 3:**
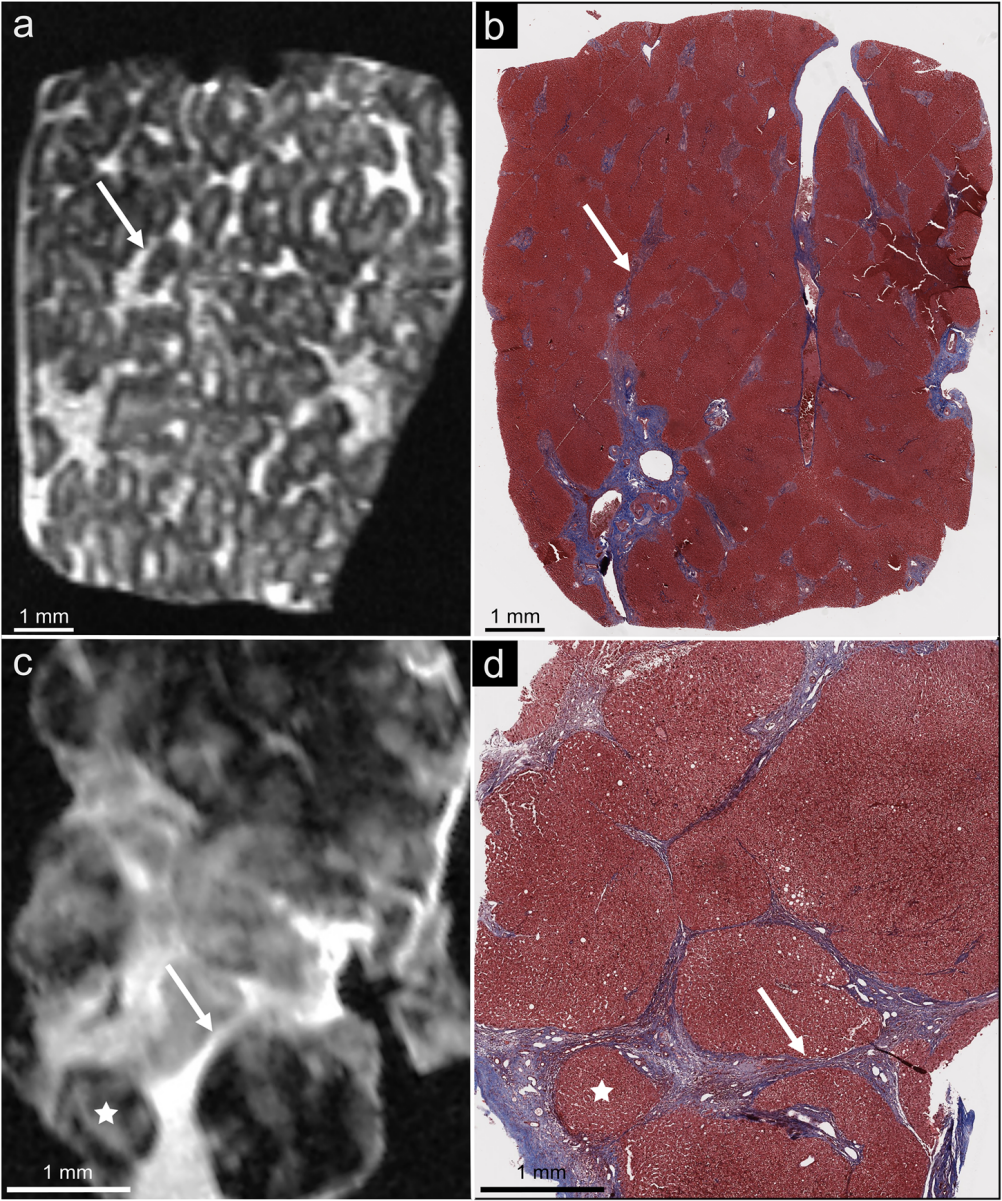
Staging discrepancy between 7-T MRI and histological reference standard. 7-T MRI (**a**) was staged F2 because of early changes in the lobular architecture and rare fibrotic septa (arrow) seen between the portal spaces, while histology (**b**) was staged F3 because of numerous fibrotic septa (arrow). **c** and **d** illustrate another example where 7-T MRI (**c**) was staged F3 due to numerous fibrotic septa (arrow) while histology (**d**) was staged F4 due to regenerative nodules (star)

The diagnostic performances of MRI for a histopathology-like diagnosis of the absence (F0) or very early (F1) fibrosis, and the presence of advanced fibrosis (F3 or F4), were excellent for both radiologists and pathologists (Table 3, Fig. 2). Radiologists achieved an 88% sensitivity (95% CI 47–100%), 100% specificity (59–100%), 100% positive predictive value (59–100%), 88% negative predictive value (95% CI 53–98%), 93% accuracy (95% CI 68–100%), 0.94 AUC (95% CI 0.69–1.0%) and 0.94 Harrell’s c-index (95% CI 0.86–1.0%). Pathologists achieved 88% sensitivity (95% CI 47–100%), 86% specificity (95% CI 42–100%), 88% positive predictive value (95% CI 53–98%), 86% negative predictive value (95% CI 48–98%), 87% accuracy (95% CI 60–98%), 0.87 AUC (95% CI 0.60–0.98%), and 0.87 Harrell’s c-index (95% CI 0.74–0.99). There were no statistically significant differences between MRI stages and pathologic gold standard stages (*p* = 0.655 for radiologists and *p* = 1.000 for pathologists). MRI correctly classified 7 of 8 patients with advanced fibrosis on pathology (1 patient was staged F2 on MRI but was F3 on histopathology). Conversely, MRI correctly excluded fibrosis (*i.e*., F0 or F1 stages) in 7 of 7 compared to histopathology. The concordance of MRI analysis with histopathology was excellent for radiologists (κ = 0.81; 95% CI 0.67–0.95) and moderate for pathologists (κ = 0.50; 95% CI 0.26–0.73) for all fibrosis stages. Figure 3 shows a discordant case between MRI assessment and histopathologic gold standard.

**Table 3:** Diagnostic performances and concordance (weighted κ) of MRI with pathologic reference standard to identify and stage hepatic fibrosis and inter-reader agreement

	Inter-reader agreement	Agreement with pathologic reference standard			
		Ratio	Accuracy	Harrell’s c-index	Weighted κ
Radiologists					
No or very early fibrosis (F0-F1)	κ = 0.32 [0.29–0.61]	7/7	93% [68–100%]	0.94 [0.86–1.00]	κ = 0.87 [0.62–1.0]
Advanced fibrosis (F3-F4)		7/8			
Pathologists					
No or very early fibrosis (F0-F1)	κ = 0.82 [0.53–1.00]	6/7	87% [60–98%]	0.87 [0.74–0.99]	κ = 0.73 [0.39–1.0]
Advanced fibrosis (F3-F4)		7/8			

Inter-reader agreement was calculated using Fleiss κ. 95% confidence intervals in squared brackets

The inter-reader agreement between the six readers was moderate (κ = 0.42; 95% CI 0.29–0.55), fair between the three radiologists (κ = 0.32; 95% CI 0.29–0.61) but almost perfect between the three pathologists (κ = 0.82; 95% CI 0.53–1.00).

## Discussion

High-field 7-T MRI demonstrated excellent performance in accurately staging liver fibrosis compared to histopathology, highlighting its potential as an innovative surrogate or complementary tool for low-magnification histology. Indeed, this study showed that the 7-T MRI can provide sufficient contrast between tissues to enable histopathology-like examination without the need for staining [7, 8]. In addition, MRI has the undeniable advantage over histology of being able to acquire images in a relatively short time with immediate image interpretation, as with any routine MRI. The specimen, which remains intact, can still be processed for pathologic examination afterward [7, 8].

Reaching very high in-plane spatial resolution (~ 75 × 75 µm^2^), close to that of low-magnification histology [20], MRI provided a completely new insight into the imaging of the liver architecture, which would not have been visible with the in-plane spatial resolution of a clinical standard MRI (~ 1 mm^2^) [21, 22]. Fibrotic changes to the liver parenchyma observed on Masson’s trichrome-stained histology slides were readily depictable on high-field MRI, appearing as T2-weighted hyperintense septa [8, 21, 22] together with fibrosis-related micro-architectural distortions at early stages [23]. While MRI has indeed a lower spatial resolution compared to histology, it provides a comprehensive assessment of liver micro-architecture, enabling the evaluation of fibrosis-related structural changes, beyond the limitations of two-dimensional histological slices. Unlike histology, which relies on two-dimensional sectioning, MRI allows for the evaluation of larger and intact tissue volumes, minimising sampling bias and preserving the native tissue structure, without the need for fixation or staining [7, 8]. Although histology remains the standard of care, it is subject to tissue-processing artifacts and sampling bias, whereas MRI provides a more comprehensive imaging.

Regarding the choice of MRI sequence, T2-weighted RARE provided an optimal balance between high in-plane spatial resolution (75 × 75 µm^2^) and contrast resolution, allowing detailed visualisation of liver micro-architecture and fibrotic tracts. Thus, the spatial resolution was optimised by adjusting acquisition parameters to maximise signal-to-noise ratio while maintaining a reasonable scanning time to prevent sample degradation.

While consensus MRI results were excellent, the inter-reader agreement between radiologists was poorer than that of pathologists. This was expected as the three radiologists had no experience with these images prior to this reading. Their only training consisted of a session based on two previously published pathological images and schematic representations, which were not representative of all stages of fibrosis. This was done to keep as many cases as possible in the final reading set. Of note, when the METAVIR score was first used by pathologists, its interobserver reproducibility was also poor [24].

The main limitation of this proof-of-concept study is the limited number of patients without any patients with F2 stage fibrosis. However, Dana et al [8] reported a case of F0 *versus* F2 accurate staging on high-field MRI, which also highlights the promising potential of the MRI-METAVIR score. Although these findings should be confirmed in a more extensive cohort, the number of expert reviewers for MRI and histology images strengthens the validity of the MRI-METAVIR score. On the other hand, the limited number of patients did not represent the full spectrum of liver conditions. Also, in case of iron overload in the liver, there is shortening of T2/T2* relaxation times, which is even worsened at 7 T, which can result in decreased signal-to-noise ratio [25]. Similarly, a fixed echo time could also influence contrast in cases of hepatic steatosis, and future studies are required to address this more specifically. In addition, although the samples were placed in Fluorinert™ Electronic Liquid FC40, a nonconductive, thermally and chemically stable fluid without ^1^H signal, the limited number of patients did not allow us to investigate the impact of the specific absorption rates and excessive heat on the samples. Another limitation is the very low number of cases (*n* = 2) in the training set, related to sample availability. Despite this limitation, the diagnostic performance of radiologists to accurately stage fibrosis did not appear to have been significantly impacted (sensitivity 88%, specificity 100%).

This study paves the way for further applications of this technique to organs other than the liver. By offering direct, pathology-like access to the micro-architecture of *ex vivo* tissue samples, high-field MRI opens up a range of new applications. This noninvasive technique broadens the scope of tissue imaging possibilities of liver, brain, brainstem, lymph nodes, or atherosclerotic plaque [7, 9, 26–28]. In surgical oncology, this technique would be particularly useful for a simplified, noninvasive, real-time analysis of surgical specimens. For instance, several attempts to assess the *ex vivo* lymph node metastatic status in breast cancer yielded promising results despite long acquisition time [7, 9]. Similarly, oncological surgeries requiring extemporaneous analysis of tissues could also benefit from this nondestructive analysis to assess tumour margins (*e.g*., partial nephrectomy, bronchial resection margins in lung cancer surgery) where the margins of a tumour could be rapidly assessed in their entirety as a three-dimensional volume without specific preparation (*i.e*., including cutting) the specimen. Additionally, volumetric images of the entire specimen could help reduce sampling errors on macroscopic evaluation of the specimens, by providing more relevant mappings than macroscopic “naked eye” analysis, to select areas to be sectioned for further histological analysis. However, the broader use of this technique as a surrogate for extemporaneous analysis has been limited by several factors, including prolonged acquisition times and the high cost and limited availability of the equipment, particularly in oncologic surgery, where perioperative decisions require rapid turnaround times. While current MRI acquisition times remain impractical for intraoperative use and/or extemporaneous examination, ongoing technological advancements, such as improved MRI coils or accelerated acquisition techniques, will hopefully significantly reduce scanning times while preserving sufficient spatial resolution and signal-to-noise ratio.

Furthermore, this method could also improve radiologists’ understanding of clinical MRI, as MRI features of diseases have often been established on an empirical basis, while the underlying pathological mechanisms inducing signal changes are not always well understood. New information, such as tissue hydration or fibrosis, may become accessible and quantifiable, in addition to the possibility of examining the entire volume of a sample without destruction [29]. In chronic liver disease, a better understanding of the normal and pathological micro-architecture of the liver parenchyma is fully in line with the current drive to develop noninvasive imaging biomarkers aiming to go beyond histopathology, aiming at replacing liver biopsies [30] but remains mostly limited to MRI elastography in fibrosis [31]. Thus, by providing unparalleled images of the liver micro-architecture, high-field MRI bridges the gap between clinical MRI and histopathology, providing a better understanding of the anatomical basis of clinical *in vivo* MRI. However, for that purpose and without being able to use a cryoprobe with clinical MRI, major technological developments are required to translate this technique, focusing on reducing breathing and motion artifacts and improving coil design. Additionally, our MRI-based results also outline potential novel research directions, such as the use of machine learning for automated fibrosis detection.

To demonstrate that MRI could be disruptively used to provide a histopathology-like examination, we needed a model allowing qualitative and quantitative analysis. Liver fibrosis appeared as an appropriate model because of the existence of widely validated histological classifications of liver fibrosis, with low subjectivity, such as the METAVIR score on Masson’s trichrome-stained sections [10–15]. The METAVIR score, validated in chronic viral hepatitis, was chosen as a uniform scoring system for all cases, regardless of the aetiology of liver disease. This represents a limitation as specific features such as perisinusoidal or perivenular fibrosis in the context of steatohepatitis were not considered. Furthermore, no patients with chronic viral hepatitis were included. Nevertheless, it enabled a fully blinded review of the cases without bias from known chronic liver disease. Further research with a larger patient cohort and various aetiologies of liver disease is required to refine our understanding of 7-T MRI liver semiology and evaluate different liver fibrosis stages, as the impact of potential confounding factors may have been overlooked in this preliminary study.

High-field 7-T MRI provides an assessment similar to low-magnification histology (1×–2×). Beyond fibrosis staging of liver tissue, MRI enables a cross-sectional volumetric exploration of the entire specimen, without cutting or destroying the sample. With short-time image acquisition and immediate image reading, high-field MRI could become a new modality for extemporaneous tissue analysis, especially in oncologic surgery. On the other hand, future research developments should focus on bridging the gap with *in vivo* MRI to impact clinical practice and provide true noninvasive microscopic histologic examination.

## Data Availability

Data are available on demand at IHU Strasbourg (corresponding author AV).

## Abbreviations

CI: Confidence interval
MRI: Magnetic resonance imaging
RARE: Rapid imaging with refocused echoes
